# Ivermectin Inhibits the Replication of Usutu Virus In Vitro

**DOI:** 10.3390/v14081641

**Published:** 2022-07-27

**Authors:** Maria Elisabeth Wald, Claudia Claus, Andrea Konrath, Hermann Nieper, Aemero Muluneh, Volker Schmidt, Thomas Wilhelm Vahlenkamp, Michael Sieg

**Affiliations:** 1 Institute of Virology, Faculty of Veterinary Medicine, Leipzig University, 04103 Leipzig, Germany; mw28guqu@studserv.uni-leipzig.de (M.E.W.); vahlenkamp@vetmed.uni-leipzig.de (T.W.V.); 2 Institute of Virology, Faculty of Medicine, Leipzig University, 04103 Leipzig, Germany; claudia.claus@medizin.uni-leipzig.de; 3 Saxon State Laboratory of Health and Veterinary Affairs, 01099 Dresden, Germany; andrea.konrath@lua.sms.sachsen.de (A.K.); hermann.nieper@lua.sms.sachsen.de (H.N.); aemero.muluneh@lua.sms.sachsen.de (A.M.); 4 Clinic for Birds and Reptiles, Faculty of Veterinary Medicine, Leipzig University, 04103 Leipzig, Germany; volker.schmidt@vogelklinik.uni-leipzig.de

**Keywords:** antiviral, bird, drug, ivermectin, Usutu virus

## Abstract

Usutu virus (USUV) is an emerging mosquito-borne arbovirus within the genus Flavivirus, family *Flaviviridae*. Similar to the closely related West Nile virus (WNV), USUV infections are capable of causing mass mortality in wild and captive birds, especially blackbirds. In the last few years, a massive spread of USUV was present in the avian population of Germany and other European countries. To date, no specific antiviral therapies are available. Nine different approved drugs were tested for their antiviral effects on the replication of USUV in vitro in a screening assay. Ivermectin was identified as a potent inhibitor of USUV replication in three cell types from different species, such as simian Vero CCL-81, human A549 and avian TME R. A 2- to 7-log10 reduction of the viral titer in the supernatant was detected at a non-cytotoxic concentration of 5 µM ivermectin dependent on the applied cell line. IC_50_ values of ivermectin against USUV lineage Africa 3 was found to be 0.55 µM in Vero CCL-81, 1.94 µM in A549 and 1.38 µM in TME-R cells. The antiviral efficacy was comparable between the USUV lineages Africa 2, Africa 3 and Europe 3. These findings show that ivermectin may be a candidate for further experimental and clinical studies addressing the treatment of USUV disease, especially in captive birds.

## 1. Introduction

Usutu virus (USUV) is an emerging arthropod-borne pathogen, responsible for considerable outbreaks in a wide range of European countries [1], leading to mass fatalities in avian populations [2,3,4,5], especially in common blackbirds [6]. As part of the Japanese encephalitis virus (JEV), antigenic complex USUV, closely related to West Nile virus (WNV), belongs to the *Flaviviridae* family within the genus *Flavivirus* [7]. First isolated from the mosquito *Culex neavei* near the Usutu River in Eswatini in South Africa in 1959 [8], it has since been found in Europe [9] and the Middle East [10]. Similar to WNV, USUV is characterized by an enzootic transmission cycle between ornithophilic mosquitoes and diverse bird species such as *Passeriformes* and *Strigiformes*, representing amplifying hosts. Clinical signs in the migratory, resident and captive avian population diverge from asymptomatic and mild to severe outcomes such as weight loss, exhaustion, ataxia and other neurological signs combined with autopsy reports of hepatosplenomegaly and necrotic lesions in heart, kidney, spleen, liver and brain [11,12,13]. In addition to the impact of USUV on wild bird populations, captive animals are also affected by this virus. For instance, canaries, finches, hawks and great grey owls housed in zoological gardens have been shown to suffer from high mortality due to USUV infections [14,15,16,17,18]. Therefore, this virus might be a risk for particular endangered avian species. Despite of the considerable clinical relevance of USUV infections in veterinary medicine recently, no approved therapeutic options are available.

Recently, drug repurposing became an attractive option for the treatment of emerging diseases, as developing new effective drugs against a particular pathogen is very time-consuming and expensive. In contrast, approved substances are easily available and their potential side effects and their pharmacokinetics are well characterized. Therefore, several compounds have already been tested for their efficacy against emerging flaviviruses. Similarly, inhibition of Zika virus (ZIKV) was shown for macrolide antibiotics such as azithromycin in vitro [19] and erythromycin estolate based on approaches in cell culture and mouse models [20]. Similarly, inhibited ZIKV replication was described in vitro for the antiparasitic drug nitazoxanide [21]. This antiprotozoal agent also displayed efficacy in JEV cell culture models and in vivo studies [22]. Other FDA-approved compounds, such as chloroquine [23], doxycycline [24,25], manidipine [26] and niclosamide [27], were reported to impair flavivirus replication in pre-clinical studies. In addition, experimental results addressing alternative properties of the old antiparasitic drug ivermectin attracted interest as a potential antiviral compound against several RNA viruses including flaviviruses, such as Dengue virus (DENV), ZIKV, Yellow Fever virus (YFV) and WNV [28]. Ivermectin, a member of the avermectin family, was discovered as a microbial fermentation product of *Streptomyces* ssp. in a soil sample in Japan in 1975 [29]. Since 1981, this macrocyclic lactone was marketed initially for veterinary usage against several parasitic infections followed by the approval for combating human onchocerciasis (river blindness) in 1987 [30] as well as strongyloidiasis and lympatic filariasis [31]. To date, no experimental data are available elucidating the antiviral efficacy of the aforementioned compounds in the context of USUV infections. Hence, this study provides in vitro data of testing the inhibitory potency of these substances against USUV replication that will inform future experimental and clinical approaches to further develop these drugs.

## 2. Materials and Methods

### 2.1. Cells

Vero (ATCC CCL-81) and A549 (ATCC CCL-185) cells were maintained in Dulbecco’s Modified Eagle Medium containing high-glucose and glutamine (DMEM GlutaMAX, Thermo Fisher Scientific, Waltham, MA, USA) supplemented with 1% (*v*/*v*) non-essential amino acids (MEM NEAA, Thermo Fisher Scientific), 1% (*v*/*v*) sodium pyruvate (Thermo Fisher Scientific), 1% (*v*/*v*) penicillin/streptomycin (P/S, Thermo Fisher Scientific) and 5% or 10% (*v*/*v*) heat-inactivated fetal calf serum (FCS, Thermo Fisher Scientific), respectively, in a humified incubator at 37 °C and 5% CO_2_. TME-R (CCL V-RIE 1164) cells displaying a finite cell line of five-day-old embryos of the Eurasian blackbird (*Turdus merula*) were grown in equal volumes of Iscove’s Modified Dulbecco’s Medium (IMDM, Thermo Fisher Scientific) and Ham’s 12 (Thermo Fisher Scientific) supplemented with 1% (*v*/*v*) penicillin/streptomycin (Thermo Fisher Scientific) and 10% (*v*/*v*) heat-inactivated FCS at 37 °C in a humified 5% CO_2_ atmosphere.

### 2.2. Viruses

USUV strains of lineage Africa 3 (NCBI accession no. KY294723.1) and Europe 3 (NCBI accession no. KY199558.1) were isolated from dead birds collected in 2019 and 2020 kindly provided by the Saxon State Laboratory of Health and Veterinary Affairs, Leipzig, Germany. In brief, a pool of organ samples (liver, kidney, brain and spleen) was homogenized in DMEM GlutaMAX supplemented with 1% (*v*/*v*) penicillin/streptomycin (Thermo Fisher Scientific) using a tissue lyser (QIAGEN, Hilden, Germany). After centrifugation (700× *g*, 5 min, 4 °C) supernatants were applied to infect Vero CCL-81 cells at 37 °C for two hours. Following incubation, cells were washed twice in PBS and infection medium (complete growth medium including 2% (*v*/*v*) FCS) was replaced. A flask of mock-infected cells served as a negative control. When over 50% of the cell monolayer showed a cytopathic effect (cell rounding and cell detachment), cell culture flasks with cells and medium were subjected to a freeze–thaw cycle at −80 °C. Cell lysates were cleared at 700× *g* for 5 min at 4 °C and supernatants were stored at −80 °C until further use. Sequencing of these USUV isolates was done by the Sanger dideoxy method and primer walking strategy [4]. The USUV strain SAAR-1776, belonging to the lineage Africa 2, was purchased from BEI Resources (Manassas, VA, USA). The infectivity titers of the applied USUV pools ranged from 6 × 10^6^ FFU/mL (SAAR-1776) to 7 × 10^7^ FFU/mL (Africa 3) and 8 × 10^7^ FFU/mL (Europe 3).

### 2.3. Viral Replication Kinetics

Viral replication dynamics and phenotypic characteristics of isolated USUV strain Africa 3 were performed in three different cell lines: Vero CCL-81, A549 and TME-R. At a density of 2 × 10^5^ cells per well, 24-well plates were seeded and incubated overnight at 37 °C and 5% CO_2_. The following day, cell monolayers were infected with a MOI of 0.01, 0.1 and 1.0. After two hours of infection, plates were washed twice in PBS and cell monolayers were covered with 1 mL infection medium per well. Supernatants were harvested every 24 h post infection, centrifuged at 500× *g* for 5 min at 4 °C and stored at −80 °C for further virus titration. All experiments were performed in independent biological triplicates.

### 2.4. Viral Titer Determination

Viral infectivity was determined in focus-forming units per ml (FFU/mL) as described previously [32]. In brief, Vero CCL-81 cells were seeded at a density of 3 × 10^4^ cells per well in 96-well plates and infected with 100 µL of 10-fold serial viral dilutions for two hours at 37 °C. Thereafter, 125 µL of 1% (*w*/*v*) carboxymethylcellulose was added to each well and plates were incubated for further three days. Subsequently, cells were fixed with 2% (*v*/*v*) formaldehyde in PBS at room temperature (RT) for 30 min and washed three times in PBS. Read-out was performed by immunofluorescence analysis (Section 2.5). Viral titers were expressed as mean focus-forming units per ml (FFU/mL), determined by manual counting of foci via immunofluorescence microscopy. All titrations were performed in biological and technical triplicates.

### 2.5. Immunofluorescence Analysis

Fixation of cell layers was performed by incubation of cell plates in 2% formaldehyde solution at RT for 30 min. A flavivirus-specific monoclonal antibody (mouse antipan-flavivirus 3571 antibody, Santa Cruz Biotechnology, Dallas, TX, USA) was diluted 1:800 in PermWash solution containing 0.1% (*w*/*v*) bovine serum albumin (BSA, Sigma-Aldrich, St. Louis, MO, USA) and 0.1% (*w*/*v*) saponine (Carl Roth, Karlsruhe, Germany) in PBS and applied for incubation at 4 °C overnight. After three washing steps, the secondary antibody goat antimouse Alexa Fluor 546 (Thermo Fisher Scientific) was diluted 1:1000 in PermWash solution containing DAPI (1 µg/mL, Carl Roth). Plates were incubated for one hour at 37 °C followed by three washing steps in PBS. Infectivity rates of cell layers were estimated semi-quantitatively by the detection of flavivirus antigen-positive cells within the cell layer compared to non-infected cells via immunofluorescence microscopy.

### 2.6. Substances

For antiviral molecule screening the compounds azithromycin, chloroquine diphosphate, doxycycline hyclate, ivermectin, manidipine hydrochloride, niclosamide and nitazoxanide were purchased from Sigma-Aldrich, chloramphenicol from Carl Roth and erythromycin estolate from Santa Cruz Biotechnology. All compounds were dissolved in dimethyl sulfoxide (DMSO, Sigma-Aldrich) at a concentration of 20 mM or 10 mM, followed by sterile-filtration (0.2 µm) and storage at −20 °C until further use.

### 2.7. Cytotoxicity Assay

Cellular toxicity of each compound was evaluated by performing ROTITEST Vital^®^ assays (Carl Roth) based on the reduction of tetrazolium salt WST-8 by living cells [33]. In brief, cells were seeded at a density of 3 × 10^4^ cells per well in 96-well plates. Serial two-fold dilutions of each compound were prepared in 100 µL triplicates and monolayers were incubated at 37 °C and 5% CO_2_ for indicated time points. Thereafter, 10 µL of the tetrazolium salt solution was added to each well for two hours followed by the measurement of the optical density at 450 nm. Infection medium containing 0.05% DMSO (*v*/*v*) served as a control, representing 100% viability of respective cells. Compounds were considered as non-cytotoxic at the indicated concentrations, displaying >90% of cell viability compared to untreated control.

### 2.8. Screening for Antiviral Activity on USUV by FDA-Approved Compounds

Due to its high permissiveness to USUV and the well-characterized phenotype [34], Vero CCL-81 cells served as a reference cell line for initial molecule screening. Cells were seeded in 24-well plates at a density of 2 × 10^5^ cells per well. Experiments were done with USUV lineage Africa 3 strain at an MOI of 0.1. Serial dilutions of each compound ranging from 5 µM to 0.156 µM in growth medium containing 0.05% (*v*/*v*) DMSO and the appropriate USUV inoculum were prepared and applied to the cells. After incubation at 37 °C in humidified 5% CO_2_ atmosphere for two hours, monolayers were washed twice in PBS to remove viral inoculum. Then, growth medium (1 mL per well) containing selected compounds at different concentrations were added (including a final DMSO concentration of 0.05 [*v*/*v*]). Cells were further incubated at 37 °C in humidified 5% CO_2_ atmosphere for three days. Infection medium comprising 0.05% (*v*/*v*) DMSO solely served as a mock infected control. After 72 h post infection, supernatants were harvested, clarified by centrifugation at 500× *g* for 5 min at 4 °C and stored at −80 °C until subjected to virus titration. Performing immunofluorescence analysis, cell monolayers were washed twice in PBS, and subsequently, were fixed applying 2% formaldehyde (*v*/*v*) in PBS at RT for 30 min followed by immunofluorescence staining, as described in Section 2.5.

### 2.9. Cell Line- and Lineage-Dependent Characterization of Antiviral Molecules

Compounds displaying a reduction of viral titer compared to the untreated virus control within non-cytotoxic concentrations during screening assay in Vero CCL-81 cells were chosen for further analysis. Therefore, two-fold serial dilutions of selected compounds reaching final concentrations ranging from 5 µM to 0.156 µM were tested against USUV lineage Africa 3 in Vero CCL-81, A549 and TME-R cells at indicated time points. Read-out was performed by two different methods: focus-forming assay for virus titer determination (Section 2.4) and immunofluorescence analysis for semiquantitative flavivirus antigen detection within the cell layer (Section 2.5). Further investigations addressing inhibitory effects against additional USUV strains in the avian cell line TME-R were performed against USUV lineage Europe 3 and USUV lineage Africa 2 at a MOI of 0.1. At 72 h post infection, virus infectivity was determined by focus-forming assay. Antigen distribution within the cell layer was evaluated by semiquantitative analysis after immunofluorescence staining. All experiments were performed in three biological and technical replicates. Indicated sampling time points for virus titrations were selected depending on replication kinetic analysis in the respective cell lines.

### 2.10. Statistical Analysis

Mean values and standard deviations (SD) were assessed in GraphPad Prism software version 9 (GraphPad Software, La Jolla, CA, USA). Then, 50% inhibitory (IC_50_) and 50% cytotoxic concentrations (CC_50_) were extrapolated from non-linear regression analysis. Selectivity indices (SI) were calculated from the quotient of CC_50_ and IC_50_. Statistical significance is indicated as n.d. (not detectable, under detection limit), * (*p* < 0.05), ** (*p* < 0.01), *** (*p* < 0.001) and **** (*p* < 0.0001) and was performed by one-way analysis of variance (ANOVA) and Dunnett’s post-hoc multiple comparisons test.

## 3. Results

### 3.1. Mammalian and Avian Cell Lines Are Susceptible to USUV Infection

**Figure 1:**
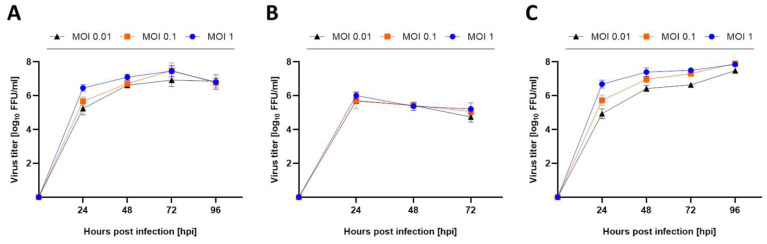
USUV replication kinetics varies in different cell lines. (**A**) Vero CCL-81, (**B**) A549 and (**C**) TME-R cell lines were infected with varying MOI doses (MOI of 0.01; MOI of 0.1; MOI of 1.0) of USUV lineage Africa 3 for 2 h. Parts from the supernatants were harvested every 24 h and viral infectivity was determined by focus-forming assay in Vero CCL-81. All experiments were performed in independent triplicates. Viral titer is depicted as mean log_10_ FFU/mL ± SD.

Viral replication dynamics of USUV strain Africa 3 indicate comparable viral titers at varying multiplicity of infection (MOI) doses and different time points in simian Vero CCL-81, human A549 and avian TME-R (Figure 1). Vero CCL-81 cells served as a reference cell line because it is widely used in the cultivation [34,35] of a broad range of flaviviruses. A549 cells, included as interferon competent cell line, displayed susceptibility to USUV, reaching the highest viral titer of approximately 6 log_10_ FFU/mL observed at 24 h post infection followed by slightly decreasing viral titers from 24 to 72 h after infection (Figure 1B). Avian cell line TME-R derived from the highly susceptible host, the Eurasian blackbird (*Turdus merula*), demonstrated permissiveness to USUV producing high viral titers (Figure 1C) similar to those from Vero CCL-81 cells (Figure 1A). In both cell lines viral infectivity sharply increased from 24 until 48 h post infection reaching a maximum of 7 log_10_ FFU/mL after 72 h. MOIs of 0.01 and 1.0 produced infections with similar kinetics and yields.

### 3.2. The FDA-Approved Compound Ivermectin Was Effective against USUV In Vitro

**Figure 2:**
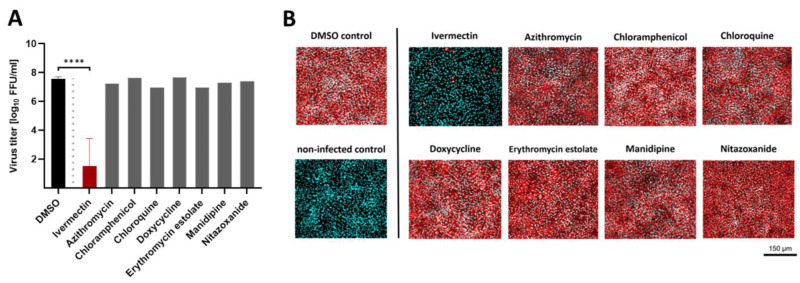
Screening of approved compounds for antiviral activity against USUV by titration of virus infectivity with a focus-forming assay. Several pharmacological molecules were screened for inhibitory efficacy against USUV lineage Africa 3 in reference cell line Vero CCL-81 at MOI of 0.1. (**A**) Extracellular infectious virus was quantified from supernatants by virus titration performing focus-forming assay. (**B**) Distribution of intracellular flavivirus antigen was evaluated by immunofluorescence analysis with a flavivirus-specific antibody. Flavivirus antigen is shown in red and nuclei stained with DAPI are shown in cyan blue. All results are demonstrated at the representative maximum concentration of 5 µM. Statistical significance is indicated as **** (*p* < 0.0001).

Nine compounds approved for several indications in human and veterinary medicine were chosen for initial antiviral screening assay against USUV lineage Africa 3 in Vero CCL-81. Prior to the evaluation of inhibitory potency, cytotoxic assays were performed in uninfected monolayers in the presence of two-fold serial dilutions of each molecule. None of the molecules, except niclosamide, displayed a cell viability lower than 90% compared to untreated control in a concentration range from 0.156 to 5 µM (details not shown). After the exclusion of cytotoxicity, inhibitory efficacy of each compound was assessed against USUV replication in Vero CCL-81 (Figure 2A, representatively shown at the maximum concentration of 5 µM). All pharmacological substances were already present during the 2 h-lasting inoculation process to address early steps of viral adherence and internalization. Later stages of viral propagation were addressed after 2 h of infection by immediate removal of viral inoculum and replacement with infection medium containing the same indicated concentration. USUV’s replication efficiency within 72 h was measured from supernatants by focus-forming assay. As shown in Figure 2A in the presence of ivermectin, a significant (*p* < 0.0001) decline of USUV’s amount of extracellular infectious virus was determined compared to untreated control. In addition, immunofluorescence analysis revealed a semiquantitative reduction of USUV’s infectivity rate within the cell layer, displaying a nearly full ablation of flavivirus antigen at 5 µM ivermectin (Figure 2B). During the screening assay, niclosamide revealed similar antiviral efficacy against USUV replication at a maximal concentration of 5 µM. Due to a decreased cellular viability (≥0.625 µM), niclosamide was excluded from this study, reaching only half maximal antiviral activity (50% inhibition compared to untreated virus control) at a low, non-cytotoxic concentration of 0.1 µM (data not shown). All other evaluated substances did not reveal a significant alteration of USUV replication in Vero CCL-81 cells and were not further investigated (Figure 2).

### 3.3. Ivermectin Inhibits USUV Infectivity in a Dose-Dependent Manner

**Figure 3:**
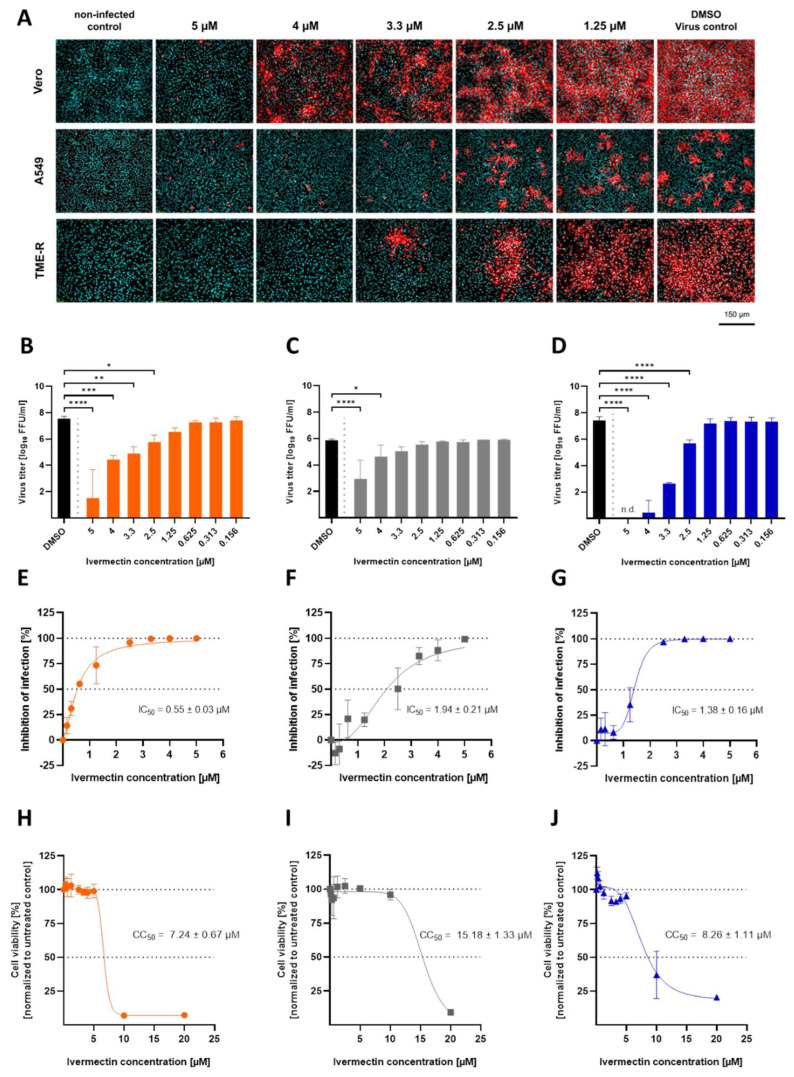
Antiviral efficacy of ivermectin against USUV in a concentration-dependent manner in several cell lines. Dose-dependent antiviral activity of ivermectin against USUV lineage Africa 3 at MOI of 0.1 was assessed in simian cell line Vero CCL-81, human cell line A549 and avian cell line TME-R. (**A**) Immunofluorescence staining of USUV-infected cells was performed in the presence of different ivermectin concentrations. Due to correlation between viral titer in the supernatant and percentage of infected cells, staining was done after 24 h in A549 and after 72 h in Vero CCL 81 and TME-R cells. Flavivirus antigen is depicted in red and cell nuclei stained with DAPI are shown in cyan blue. (**B**–**D**) Quantification of extracellular infectious USUV particles released from Vero CCL-81 (**B**), A549 (**C**) and TME-R (**D**) was evaluated by virus titration. (**E**–**G**) Half maximal inhibitory concentrations (IC_50_) of ivermectin were calculated in Vero CCL-81 (**E**), A549 (**F**) and TME-R (**G**) cells. Inhibition of infection was calculated by titration of supernatants from untreated USUV control in comparison to ivermectin treated panels. Data represent mean values ± SD from independent triplicates. (**H**–**J**) Viability of different cell lines in the presence of ivermectin. Cytotoxicity of ivermectin was measured in two-fold serial dilutions in cells lines Vero CCL 81 (**H**), A549 (**I**) and TME-R (**J**), applying a WST-8 tetrazolium salt system. Results are depicted as mean percentages ± SD of viable cells in comparison to the untreated control. Experiments were performed in independent triplicates. CC50 values were calculated by non-linear regression analysis performed in GraphPad Prism software 9. Statistical analysis was performed by ANOVA and Dunnett’s post-hoc multiple comparisons test evaluating IC_50_ values by non-linear regression analysis and indicating statistical significance as n.d. (not detectable, under detection limit), * (*p* < 0.05), ** (*p* < 0.01), *** (*p* < 0.001) and **** (*p* < 0.0001).

For the evaluation of inhibitory doses, immunofluorescent antigen staining of the cell layer and viral titer determination from supernatants of USUV-infected (lineage Africa 3) cells were performed. Ivermectin at a concentration of 5 µM led to almost no intracellular antigen signal detection by immunofluorescence (Figure 3A). In addition, viral titration of the supernatants displayed a highly significant (*p* < 0.0001) reduction of extracellular viral infectivity (Figure 3B–G). Furthermore, inhibition of USUV replication at declining concentrations of ivermectin (<5 µM) revealed reduced amounts of USUV antigen-positive cells as low as 2.5 µM of ivermectin in all selected cell lines (Figure 3A). Comparable results were detected by quantification of supernatant virus infectivity titers, although some cell type-specific differences were uncovered (Figure 3B–G). Whereas the USUV titer decreased significantly by 10–100 log_10_ FFU/mL in the presence of 2.5 µM ivermectin in Vero CCL-81 (Figure 3B) and TME-R (Figure 3D), a significant reduction of viral load was only present at the concentrations of 5 µM (*p* < 0.0001) and 4 µM (*p* = 0.05) ivermectin in A549 cells (Figure 3C,F). A decline of viral titer was most prominent in Vero CCL-81 cell line and was accompanied by reduced infectivity rate estimated by immunofluorescence analysis at ivermectin concentrations as low as 1.25 µM. The calculation of the half maximal inhibitory concentration (IC_50_) against USUV infection in the presence of ivermectin was lowest in Vero CCL-81 (Figure 3E) followed by TME-R (Figure 3G) and A549 (Figure 3F). Based on non-linear regression analysis the IC_50_ of ivermectin against USUV lineage Africa 3 was evaluated as 0.55 ± 0.03 µM in Vero CCL-81 (Figure 3E), 1.94 ± 0.21 µM in A549 (Figure 3F) and 1.38 ± 0.16 µM in TME-R cells (Figure 3G) from three independent experiments.

Cytotoxicity was evaluated based on formazan formation from tetrazolium salt WST-8 by enzymatic processes in living cells (Figure 3H–J). Cell line-dependent CC_50_ values of ivermectin resulted in 7.24 ± 0.67 µM in Vero CCL-81 (Figure 3H), 15.18 ± 1.33 µM in A549 (Figure 3I) and 8.26 ± 1.11 µM in TME-R (Figure 3J) and were determined from three independent experiments.

The calculated CC_50_ and IC_50_ values for each cell line were used to determine the in vitro selectivity index (SI) for ivermectin (Table 1). The SI values were determined as follows: 13.16 in Vero CCL-81, 7.82 in A549 and 5.99 in TME-R cells (Table 1).

### 3.4. Ivermectin Displays Antiviral Efficacy against Other USUV Strains In Vitro

**Figure 4:**
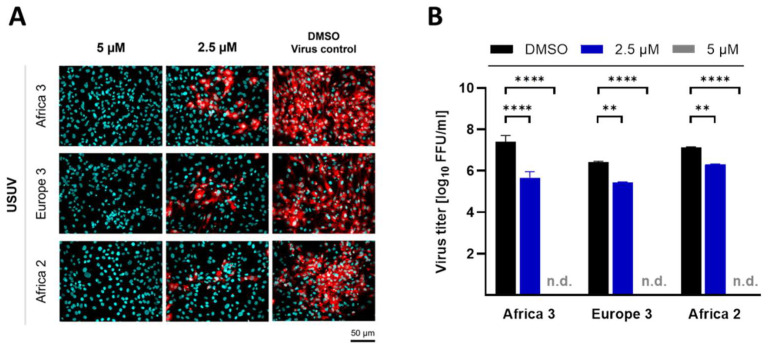
Antiviral activity of ivermectin against several USUV strains in vitro. TME-R cells were infected with USUV lineages Africa 3, Europe 3 and Africa 2 at MOI of 0.1 in the presence of selected ivermectin concentrations. Readout was performed after 72 h post infection. (**A**) Immunofluorescence staining of USUV-infected cells was performed in the presence of selected ivermectin concentrations. Flavivirus antigen is depicted in red and cell nuclei stained with DAPI are shown in cyan blue. (**B**) Virus titer was determined from supernatants of infected cells treated with 2.5 µM and 5 µM of ivermectin compared to vehicle control (DMSO). N.d. = not detectable. Data represent mean values ± SD from three independent experiments. Statistical analysis was performed by ANOVA and Dunnett’s post-hoc multiple comparisons test indicating statistical significance as n.d. (not detectable, under detection limit), ** (*p* < 0.01) and **** (*p* < 0.0001).

To exclude a lineage-specific antiviral effect of ivermectin against USUV replication of strain Africa 3, investigations of further USUV lineages were conducted at defined concentrations of ivermectin. For this purpose, TME-R cells were infected with USUV strains (MOI of 0.1) representing the lineages Europe 3 and Africa 2 in the presence of selected concentrations of ivermectin. Impairment of viral replication was determined by immunofluorescence staining of USUV antigen-positive cells and by quantification of infectious viral particles in the supernatant after 72 h of infection. Similar to the antiviral effects observed against lineage Africa 3 (Figure 3A–G), USUV replication of strain Europe 3 and Africa 2 was reduced by ivermectin as demonstrated by the complete ablation of flavivirus antigens in the cells (Figure 4A) and a highly significant (*p* < 0.0001) loss of viral titer at 5 µM of ivermectin (Figure 4B). Furthermore, a highly pronounced antiviral effect was detected at 2.5 µM of ivermectin, as indicated by a semiquantitative decline of USUV’s infectivity rate in immunofluorescence analysis (Figure 4A) and a significant (*p* < 0.01) reduction of replication detected by lower yields of infectious virus (Figure 4B).

## 4. Discussion

Severe clinical manifestations and high mortalities in avifauna emphasize the need for therapeutic options against USUV. For this reason, we evaluated nine FDA-approved compounds for their antiviral activity against USUV in vitro and identified ivermectin’s inhibitory potency against several USUV lineages in cell culture.

The selection of these pharmacological molecules formerly approved for the treatment of several infectious and non-infectious human and animal diseases included recently reported substances, which were tested for their antiviral effects. These compounds were evaluated as drug repurposing candidates against flaviviruses in vitro and partly in clinical trials [36,37,38,39]. Thus far, none of them were tested for antiviral activity against USUV. Therefore, we evaluated these compounds in an USUV in vitro replication model applying the flaviviral reference cell line Vero CCL-81 [34]. Except for ivermectin, none of them had any measurable impact on extracellular viral titer or intracellular viral infectivity in our cell culture model. This observation might be explained by the fact that the described inhibitory effects are specific for ZIKV, DENV or JEV. Furthermore, antiviral efficacy differs between cell types, as was exemplified for the differential inhibition of DENV type 2 by chloroquine in Vero and C6/36 cell lines [40].

In contrast to the aforementioned compounds, the broad-spectrum antiparasitic macrocyclic lactone ivermectin exhibited a dose-dependent, but cell type- and virus lineage-independent antiviral effect against USUV. To rule out a Vero- and TME-R-specific effect of ivermectin, we included the widely used lung epithelial cell line A549 into our study. Due to its completely different origin, this human cell line is unrelated to Vero and TME-R cells. Moreover, A549 cells provide a competent type I IFN system, which is in contrast to the flaviviral reference cell line Vero [34], which is lacking IFN type I genes [41,42]. Furthermore, avian TME-R cells were analyzed because they are a cell culture model for one of the main clinically affected hosts, the Eurasian blackbird [13]. The significant loss of viral infectivity at 5 µM of ivermectin over at least three (A549) and seven (TME-R) log_10_ steps of FFU/mL supports further transfer of ivermectin into clinical studies. Calculated IC_50_ values against USUV lineage Africa 3 in the low micromolar range (0.54 µM in Vero CCL-81; 2.02 µM in A549; 1.4 µM in TME-R) are comparable with the determined efficacy for related flaviviruses in different cell lines tested [28]. The inclusion of further USUV lineages such as Europe 3 and Africa 2 demonstrated ivermectin’s independence from strain-specific inhibitory effects in avian TME-R cells. Similar results were observed for several DENV serotypes displaying comparable IC_50_ values of ivermectin in the low micromolecular range when tested in vitro [43].

It is important to mention that the observed low micromolar IC_50_ values are markedly higher then serum concentrations which were measured in ivermectin-fed chickens [44]. In the aforementioned study, chickens received medicated feed including 200 mg ivermectin per kg. On average, every animal received approximately 30 mg ivermectin daily, resulting in a maximum ivermectin serum concentration of 155 ng/mL (corresponding to 0.17 µM). However, ivermectin was administered orally and the chickens received the food-free of choice, so it is not clear how much of the drug was absorbed. In another study, it was shown recently that oral treatment resulted in a low plasma bioavailability in laying hens in contrast to subcutaneous and intravenous routes, which were much more effective [45]. Another study showed that falcons can be treated intramuscularly with 5 mg/kg without having any clinical side effects [46]. Unfortunately, the authors did not measure serum concentrations of ivermectin in the treated animals. Moreover, pure translation of in vitro to in vivo data appears insufficient due to ivermectin’s complex antiviral mechanisms. Apart from a direct antiviral capacity against YFV, DENV and WNV relying on targeting the non-structural protein 3 [47], indirect, host-dependent impacts of ivermectin are assumed. The functions of the non-structural replication proteins of members of the genus Flavivirus are conserved and ivermectin may utilize the same antiviral mechanisms against USUV as previously reported for other flaviviruses. In addition, another possible mechanism for the observed USUV inhibition might be due to importin-associated blocking of viral protein translocation into nucleus [48]. In DENV-infected cells, this translocation is associated with non-structural (NS) protein 5 and its RNA polymerase activity [43]. Furthermore, proposed stimulation of the immune system by ivermectin [49] might contribute to the assumption that even a low dose of ivermectin enhances host-specific antiviral processes combating virus replication [50,51]. Additionally, accumulation of ivermectin is described for some organs, especially for the liver and for adipose tissues [52]. Furthermore, despite the very high in vitro antiviral concentrations (>50 µM) necessary for the inhibition of porcine circovirus 1 in PK-15 cells, in vivo efficacy in piglets was confirmed in decreasing genomic virus load in several tissues [53]. Hence, the comparison of calculated IC_50_ values in vitro with plasma concentration levels in vivo may result in an underestimation the compound’s antiviral potency.

Focusing on former studies in vivo ivermectin’s potent antiviral capacity was demonstrated against Pseudorabies virus in a mouse model [54]. In contrast, lethal outcome of ZIKV-infected immunodeficient mice was observed despite of ivermectin treatment [55]. In parallel, results of several clinical studies addressing the antiviral efficacy of ivermectin against SARS-CoV-2 revealed inconclusive data [56,57,58,59]. Nevertheless, lacking evidence of ivermectin’s antiviral efficacy for particular viruses, notably in humans and immunodeficient mouse models, has to be distinguished from approaches analyzing feasible inhibitory activity in avian species due to possible different pharmacokinetics. Anecdotic reports implied improved clinical outcome in confirmed cases of WNV or USUV disease in owls and hawks due to ivermectin treatment [60,61]. On the other hand, these two studies lack a non-treated control group, the quantity of involved animals were very low and the animals were simultaneously treated with fluorchinolones and voriconazol.

In consideration of the aforementioned inconsistent data regarding ivermectin’s antiviral efficacy under in vivo conditions, randomized double-blinded animal studies are required to clarify ivermectin’s potential for drug repurposing against USUV. This would be especially useful for captive birds belonging to the *Passeriformes* and *Strigiformes*, as some of these species suffer from severe or fatal outcomes in USUV infections. 

## Figures and Tables

**Table 1:** Calculations of IC_50_, CC_50_ and SI of ivermectin against USUV replication in vitro. Conventional parameters due to non-linear regression analysis performed by GraphPad Prism software 9 yielded inhibitory efficacy of ivermectin against USUV lineage Africa 3 (MOI of 0.1) and the corresponding cytotoxicity in Vero CCL-81, A549 and TME-R. Means ± SD of IC_50_ and CC_50_ values in addition to elaborations of the SI as the quotient of CC_50_ and IC_50_ demonstrating the ratio between cytotoxicity and antiviral activity are depicted and referred to data from at least three independent experiments.

Cell Line	Inhibition Efficacy IC_50_ (µM)	Cell Viability CC_50_ (µM)	Selectivity Index SI (CC_50_/IC_50_)
Vero CCL-81	0.55 ± 0.03	7.24 ± 0.67	13.16
A549	1.94 ± 0.21	15.18 ± 1.33	7.82
TME-R	1.38 ± 0.16	8.26 ± 1.11	5.99

## Data Availability

The raw data supporting the conclusions of this article will be made available by the corresponding author without undue reservations.
